# A dataset for fine-grained seed recognition

**DOI:** 10.1038/s41597-024-03176-5

**Published:** 2024-04-06

**Authors:** Min Yuan, Ningning Lv, Yongkang Dong, Xiaowen Hu, Fuxiang Lu, Kun Zhan, Jiacheng Shen, Xiaolin Wu, Liye Zhu, Yufei Xie

**Affiliations:** 1 School of Information Science and Engineering, Lanzhou University, Lanzhou, Gansu 730000 China; 2 State Key Laboratory of Herbage Improvement and Grassland Agro-ecosystems; Key Laboratory of Grassland Livestock Industry Innovation, Ministry of Agriculture and Rural Affairs; Engineering Research Center of Grassland Industry, Ministry of Education; College of Pastoral Agriculture Science and Technology, Lanzhou University, Lanzhou, 730000 China; 3 George R. Brown School of Engineering, Rice University, Houston, USA

**Keywords:** Plant breeding, Agroecology

## Abstract

The research of plant seeds has always been a focus of agricultural and forestry research, and seed identification is an indispensable part of it. With the continuous application of artificial intelligence technology in the field of agriculture, seed identification through computer vision can effectively promote the development of agricultural and forestry wisdom. Data is the foundation of computer vision, but there is a lack of suitable datasets in the agricultural field. In this paper, a seed dataset named LZUPSD is established. A device based on mobile phones and macro lenses was established to acquire images. The dataset contains 4496 images of 88 different seeds. This dataset can not only be used as data for training deep learning models in the computer field, but also provide important data support for agricultural and forestry research. As an important resource in this field, this dataset plays a positive role in modernizing agriculture and forestry.

## Background & Summary

The continuous development of deep learning technology cannot be separated from the support of data, which is becoming increasingly important. In the field of computer vision, data is the foundation, image acquisition is particularly important. Imagenet^1^, as a very important data set in computer vision, was created by Professor Li Feifei and has been the benchmark for evaluating image classification algorithms. This data set has greatly facilitated the development of the image classification task, with tens of thousands of images examined. With the development of fine-grained classification tasks, various data sets for fine-grained image classification tasks continue to appear. Stanford Cars^2^ is a fine-grained dataset for car classification, consisting of 16,185 images of 196 different types of car images. Similarly, FGVC-Aircraft^3^ is a fine-grained classified aircraft image dataset with 10,200 aircraft images of 102 different aircraft models. Cub200^4^ is a fine-grained dataset proposed by the California Institute of Technology in 2010, containing 200 bird species with a total of 11,788 bird images for training neural network models.

In agriculture, datasets for seed classification have been lacking. Datasets in agriculture are mainly plant leaves or plant images, such as PlantCLEF^5^, VNPlan t^6^, BJFU100^7^ and other data sets. The VNPlant dataset consists of a total of 20,000 images of 200 different labeled Vietnamese medicinal plants (Vnplant-200). The dataset is available in 256 × 256 and 512 × 512 pixel versions. The training set contains 12,000 images and the rest for the test set. The BJFU100 dataset is the first data set of plant images in natural scenes collected by mobile phones. It was proposed by China Agricultural University and contains 100 plant species on campus in Beijing, with a total of 10,000 images. These datasets are mostly images of plant leaves and are mainly used for plant classification and pest identification applications.

In terms of seed identification, there are two main problems. One is that the dataset in seed does not match the data needed for deep learning. For example, for some seed datasets, one kind of seed has only one or several images, so it is difficult to train the neural network and apply it to the task of seed image classification. Second, Some seed datasets have not been published, which makes it difficult to use the data. At the same time, the format and quantity of data sets are inconsistent, which also makes it difficult to use data sets. At the same time, some seed datasets only target a certain class or family of seeds, and the seed category and number are small, which can not achieve a good classification of multiple seed images. Therefore, in order to solve the problem of shortage of data sets, this paper establishes a data set for seed classification.

Because of the characteristics of seeds, it is difficult to obtain images. Due to the small size of the seeds, image acquisition requires certain equipment and acquisition methods. Due to the different growth periods and storage environments of seeds, the same kind of seeds have great differences in shape, size and other aspects. At the same time, due to the extremely rich seed texture characteristics, different shooting conditions will cause the difference in seed images. In addition, due to the different smoothness and color of the seed surface, different lighting conditions will also make certain differences in the image. RGB and spectral images are commonly used in seed classification images. Acquiring a spectral image requires certain equipment. At the same time, the acquisition of RGB images is convenient with lower cost and wider application range, which is also more conducive to the further expansion of datasets in the later period. Therefore, this study provides a mobile phone and studio-based image acquisition equipment. A total of 88 different types of seed images were collected. After correlation processing and manual screening, a LZUPSD dataset containing 4496 seed images was established.

Advances in deep learning have led to several real-world domains, such as medical analytics^8^, autonomous driving^9^, and agriculture^10^. The success of deep learning is largely due to abundant computing resources, well-designed network architectures, and large-scale datasets. However, to the best of our knowledge, very few seed datasets are publicly available. Most of its publicly available datasets are plant species, pest and disease datasets^11,12^. Many scholars have also done a lot of research on seed image classification. Granitto PM *et al*.^13^ created a seed dataset in 2002 containing 57 categories of weed seeds and 3163 color images to explore seed classification methods. In 2005, the team^14^ further built a large dataset with 236 different seed species. The dataset contained 10,310 color images of seeds. Luo *et al*.^15^ used cameras, conveyor belts, and other devices to collect seed images and build a seed dataset. Based on the establishment of a weed seed image acquisition system, they quickly and completely segmented a single weed seed image, and a total of 140 weed seed species and 47696 foreign body samples were retained. Giselsson *et al*.^16^ presented a dataset containing images of about 960 unique plant species from 12 different growth stages. It contains annotated RGB images with a physical resolution of about 10 pixels per millimeter. There is a risk of invasive alien species in the transboundary transport and logistics of exotic plant seeds transported with imported goods. It is very important to establish strict inspection and quarantine procedures, actively manage and control the import and export of foreign seeds. There is therefore an urgent need for a rapid method of identifying exotic plant seeds. Yang *et al*.^17^ built a database of 3,000 seed images of 12 invasive plant species. Mast seeding is a highly variable and spatially synchronous method of seed production for plant populations. Different seed crop production is often weather-related, so whether global climate change changes the variability of harvests or the scale of harvest events is significant. Li *et al*.^18^ compiled 1,086 plant seed production data sets from around the world from 1900 to 2014.

Seed classification identification is of great significance for limiting weeds, preventing invasion of alien species, and identifying seed quality^19^. Most weeds spread in the form of seeds, which will cause serious harm to agricultural production, the ecological environment and biodiversity. Meanwhile, seed classification plays a very important role in identifying seed quality and sowing^20,21^. With the continuous development of computer vision, deep learning technology is more and more applied in agriculture and forestry, such as plant classification^22,23^, plant pest identification, etc. In 2019, Liao *et al*.^24^ used hyperspectral and transfer learning techniques to identify corn seeds through a pre-trained VGG19 model. 2020Peng Lei *et al*.^25^ maize seed vigor estimation and germination prediction using hyperspectral and convolutional neural networks. Some datasets have some problems, such as insufficient data, not being publicly available, and the cost of hyperspectral data acquisition is high. The use of mobile phone image acquisition is simple, fast, low cost, conducive to the subsequent expansion of the dataset and other operations.

## Methods

In this section, we present seed identification as a challenge related to the computer vision task, describe the equipment used for the acquisition, and the data processing process, and finally present our dataset. This chapter mainly discusses the whole process and method of making seed dataset. In the chapter of Technical Validation, some mainstream deep learning models are used to further verify the validity of the dataset established in this paper.

### Challenge

In recent decades, seed identification, as a routine but vital part of seed determination, has often been performed manually. Careful examination of each seed in a batch of seed samples and determination of grain subtypes is a complex and extensive task. With the continuous development of computer vision, deep learning technology is increasingly applied in the field of agriculture and forestry, such as plant classification, plant pest and disease identification, etc. It is of practical significance to apply deep learning to seed classification and recognition. Due to the mildew of the seeds, pictures taken by mosquitoes after being bitten will be different from the shape of the seeds themselves. In addition, many weed seeds are very small, so it is difficult to take pictures of their unique characteristics, which makes it difficult to identify seeds. Fine-grained classification is a hot research direction in recent years. In order to distinguish pictures with particularly small differences in shape, it pays attention to more subtle features. Seeds not only have very small differences within classes, but can also be very similar between classes, which poses a bigger problem with seed classification and recognition.

Objects for fine-grained classification tasks usually have similar appearance characteristics in terms of shape, color, and texture. For example, some seeds, although belonging to different classes, have only slight differences in texture. However, there are obvious differences between normal and mildewed or mosquito-bite grains, and the seeds can be quite different at different stages of the same plant’s growth. These differences in appearance are even greater among seeds of the same species than among seeds of different species. These problems make it difficult to classify and identify seeds.

### Overall scheme

**Fig. 1:**
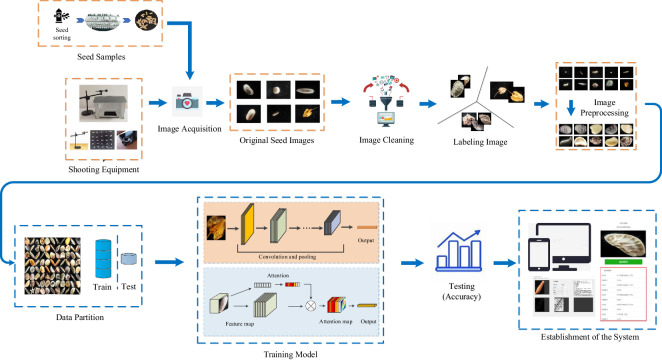
The overall seed dataset scheme. After sampling the seeds in the seed bank using a collection device and collecting them, the original seed image data is obtained. Then, the original seed image is data cleaned, classified, labeled, and cropped to obtain the seed dataset. Using seed datasets for deep learning model training can train models for seed classification and recognition.

After the seeds are collected, they must be screened with special equipment. They are screened and packaged in bottles labeled and stored at low temperatures. In the process of establishing the seed dataset, we take the seeds and place them on a black mat. A smartphone is attached to a stand and the seeds are photographed. After shooting, the image will be cleaned to remove images that do not meet the specifications. After that, because the seeds are too small, the image needs to be centered. The captured seed images are then placed in different folders according to categories to form a seed dataset. Based on the established seed dataset, each seed image type is divided into a training set and a test set at a ratio of 4:1. The training set is sent to the neural network for learning. After learning, test sets are used to test the learned network model. Finally, the model trained with the seed dataset is used as the back-end system mounted on the seed recognition and classification system. Users can take photos and upload pictures by mobile phone or computer to obtain seed recognition and classification results. The whole process of the scheme is shown in Fig. 1.

### Data acquisition equipment

**Fig. 2:**
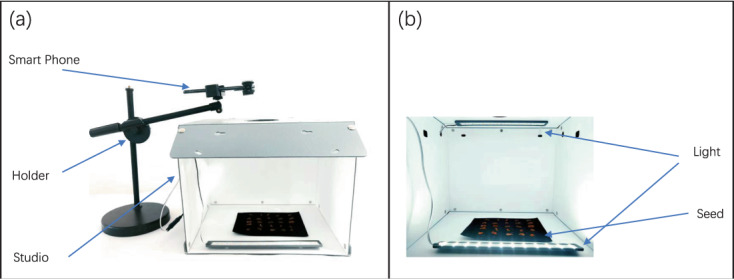
Seed data acquisition equipment. (**a**) the overall structure of the camera phone holder and studio. (**b**) the situation inside the studio.

**Fig. 3:**
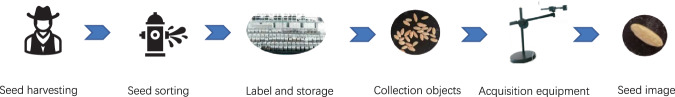
Flow chart of seed image acquisition. After collecting seeds, specialized equipment is needed to select them based on factors such as weight, and then label and store them. Filter the seeds stored in the seed bank and use a shooting device to collect images to establish a seed dataset.

Seed samples are required to obtain seed images. The seeds collected this time are provided by the Grass Lawn Seed Quality Supervision, Inspection and Testing Center (Lanzhou) of the Ministry of Agriculture. Various seed classification methods use hyperspectral images, but obtaining hyperspectral images requires specific equipment, is costly, and cannot be widely applied. The images used in daily life are mostly RGB images obtained through devices such as cameras or mobile phones. Therefore, considering the simplicity and applicability of the collection method, in order to adapt the dataset to more real-life scenarios, this article uses mobile phones to capture seed images. Figure 2a shows the overall architecture of the shooting, including the phone holder, smartphone, and filming studio. The phone model used is Xiaomi Mix2, with a rear camera of 12 million pixels; The aperture is f/2.0, and the sensor type is CMOS. This phone is equipped with a professional distortion free macro lens, with an effective focusing range of 20 mm–70 mm. Figure 2b shows the interior of the filming studio. The studio is a double-layer PP plastic opaque box with a size of 330× two hundred and sixty ×260 mm; The stage is used to place actual plant seeds, and a layer of black PVC cloth is laid on the contact surface with the seeds. The resulting image is a black background, which can enhance the color contrast between the seeds and the background; The light source consists of two LED panels with a color temperature of 5500 K, with an input voltage of 100 V to 240 V, an output voltage of 12 V, and a brightness of 8500LUX. The illumination is sufficient to cover the entire seed and the light is uniform; When collecting images, place the seeds at the center mark of the stage. In order to ensure the smooth and rapid progress of the shooting and avoid external environmental interference, the shooting is carried out in the studio. There is a circular opening above the studio that serves as a hole for taking photos with a mobile phone. Meanwhile, place the phone on the stand so that it is positioned above the circular hole. The seeds will be placed on the blackout cloth, placed in the studio, and then adjusted in place. The assembled phone and macro lens are placed on a stand to adjust their height and angle to align with the circular hole above the studio, making the image clear. The collected image size ranges from 50 K to 1 M, with a size of 1600 × Around 1200. To get the data efficiently, we set up a data handler, as shown in Fig. 3.

### Data acquisition and processing

**Fig. 4:**
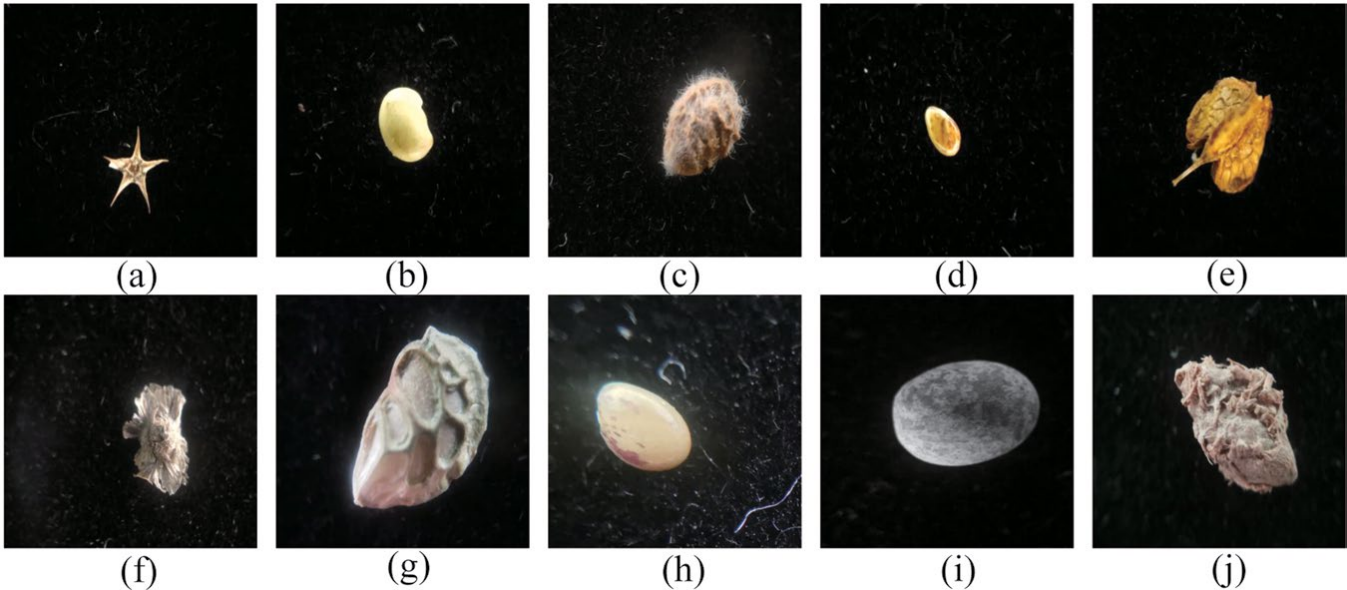
Original seed image. (**a**) *Bassia dasyphylla*, (**b**) *Halimodendron halodendron*, (**c**) *Hedysarum scoparium*, (**d**) *Lespedeza bicolor*, (**e**) *Rumex patientia*, (**f**) *Salsola tragus*, (**g**) *Glycyrrhiza glabra*, (**h**) *Lespedeza daurica*, (**i**) *Vicia sativa*, (**j**) *Calligonum mongolicum*.

**Fig. 5:**
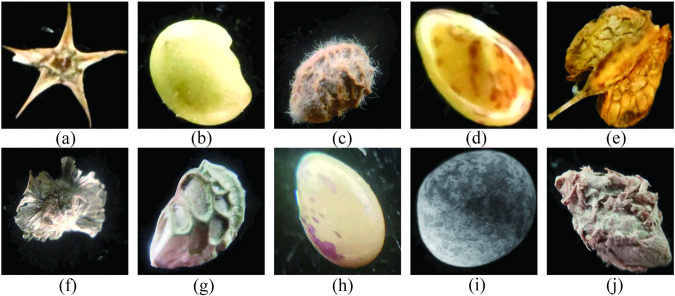
The pre-processed seed image. (**a**) *Bassia dasyphylla*, **(b**) *Halimodendron halodendron*, (**c**) *Hedysarum scoparium*, (**d**) *Lespedeza bicolor*, (**e**) *Rumex patientia*, (**f**) *Salsola tragus*, (**g**) *Glycyrrhiza glabra*, (**h**) *Lespedeza daurica*, (**i**) *Vicia sativa*, (**j**) *Calligonum mongolicum*.

Due to the small size of the seeds, they are susceptible to interference such as background. Therefore, in order to avoid the interference of complex background on seed image classification, the collected seed samples were evenly placed on the black blackout cloth. The seeds will be arranged in 5 × 5 rectangles for easy shooting. Place it in the studio, position the blackout cloth and phone so that the seed image in the phone becomes clear, and then press the Shoot button to take the photo. The lighting was also adjusted, and three different lighting conditions were used to simulate the influence of different lighting conditions on the seed image in the real scene. At the same time, the position and angle of the seed were adjusted to obtain a richer seed image. After taking seed images, seeds should be classified and stored according to the requirements of the Quality Supervision, Inspection and Testing Center of Grass and Turf Grass Seeds of the Ministry of Agriculture (Lanzhou). The size of the captured image ranges from 50k to 1 M, and the size is about 1600 × 1200. Due to the small size of the seed, most of the original image obtained by shooting is the background, which will affect the result to some extent. In order to obtain a clearer seed image, it is necessary to crop the seed image to some extent. And the seed image is cropped by downsampling and other means to remove the background part and preserve the seed part as much as possible, so that the image is mostly seed. The size of the cropped image is 192 × 272, and the image contains almost only the seed portion. After image collection and processing is completed, images are manually screened, unavailable images are deleted, and classification and evaluation are performed simultaneously. Figures 4, 5 show the images before and after processing. To avoid data imbalance, image data was further screened to keep the number of images per seed at about 50.The names of the 88 seeds included in this dataset are shown in Table 1.

**Table 1:** Names of 88 different categories of seeds contained in the dataset.

number	species	number	species	number	species
1	*Lycium chinese*	2	*Caragana microphylla*	3	*Corethrodendron fruticosum var. mongolicum*
4	*Corethrodendron scoparium*	5	*Medicago lupulina*	6	*Leymus chinensis*
7	*Thlaspi arvense*	8	*Elymus nutans*	9	*Vicia villosa*
10	*Poa annua*	11	*Apocynum venetum*	12	*Melilotus officinalis*
13	*Plantago major*	14	*Cynanchum acutum*	15	*Cynanchum acutum*
16	*Astragalus laxmannii*	17	*Leonurus japonicus*	18	*Trifolium repens*
19	*Apocynum pictum*	20	*Thermopsis lanceolata*	21	*Limonium aureum*
22	*Vicia sativa*	23	*Atriplex sibirica*	24	*Achnatherum splendens*
25	*Halogeton arachnoideus*	26	*Halostachys caspica*	27	*Hippophae rhamnoides*
28	*Descurainia sophia*	29	*Avena sativa*	30	*Lolium perenne*
31	*Euphrasia pectinata*	32	*Amorpha fruticosa*	33	*Buddleja alternifolia*
34	*Artemisia sphaerocephala*	35	*Picea asperata*	36	*Rumex patientia*
37	*Lespedeza bicolor*	38	*Hyoscyamus niger*	39	*Saposhnikovia divaricata*
40	*Anemone rivularis*	41	*Artemisia ordosica*	42	*Artemisia desertorum*
43	*Haloxylon ammodendron*	44	*Caragana korshinskii*	45	*Nitraria tangutorum*
46	*Festuca rubra*	47	*Clematis fruticosa*	48	*Zygophyllum xanthoxylon*
49	*Halimodendron halodendron*	50	*Caragana liouana*	51	*Agropyron mongolicum*
52	*Reaumuria songarica*	53	*Triticale*	54	*Puccinellia distans*
55	*Bromus inermis*	56	*Achnatherum inebrians*	57	*Cosmos bipinnatus*
58	*Nitraria sibirica*	59	*Kochia scoparia*	60	*Hordeum vulgare*
61	*Medicago sativa*	62	*Pisum sativum L*	63	*Agropyron cristatum*
64	*Puccinellia tenuiflora*	65	*Calligonum mongolicum*	66	*Elymus pendulinus subsp. pubicaulis*
67	*Elaeagnus angustifolia*	68	*Oxytropis bicolor*	69	*Caryopteris mongholica*
70	*Elymus sibiricus*	71	*Salsola tragus*	72	*Coronilla varia*
73	*Iris lactea*	74	*Lespedeza daurica*	75	*Corethrodendron scoparium*
76	*Thinopyrum ponticum*	77	*Calligonum mongolicm Turcz*	78	*Agriophyllum squarrosum*
79	*Amygdalus mongolica*	80	*Peganum harmala*	81	*Lycium ruthenicum*
82	*Setaria viridis*	83	*Bassia dasyphylla*	84	*Astragalus melilotoides*
85	*Corethrodendron lignosum*	86	*Stipa bungeana*	87	*Ligularia virgaurea*
88	*Rumex nepalensis*				

## Data Records

In order to promote research progress in our field and enhance the visibility of our research, we share our seed dataset LZUPSD^26^ with other researchers at: 10.6084/m9.figshare.24552394.v1. We are in the process of adding many more seeds to expand our database.

**Fig. 6:**
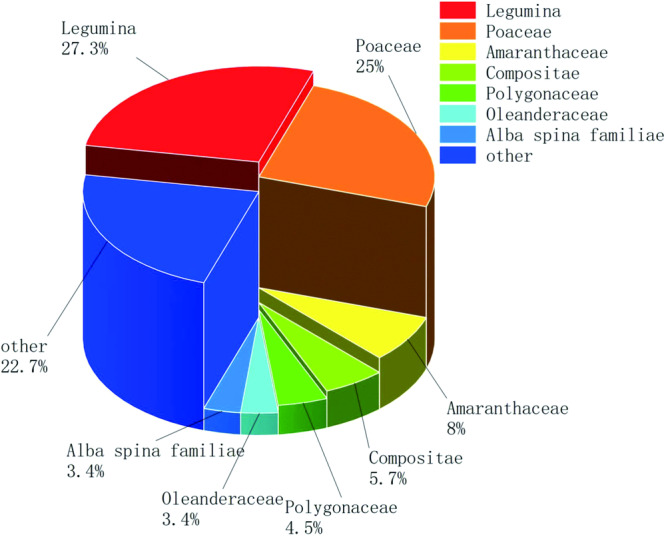
Distribution of seed family genera. In the 88 different types of seed datasets collected in this article, the largest species is legumes with 24 species, followed by the Gramineae family with 22 species, and other families and genera contain fewer species.

**Fig. 7:**
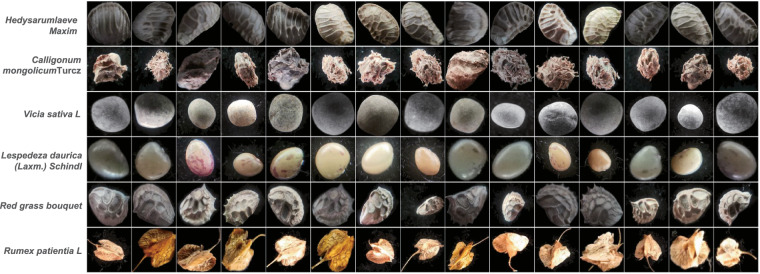
Seed image display. Each row is of the same species, and the seeds of the same species may vary slightly due to differences in growth cycle and environment. And the images taken from different angles will also vary.

After pruning and screening, 4,496 images were left, with about 50 images for each seed. Data were divided into training and test sets by 4:1. In the end, each seed image of the training set contains about 40 images, totaling 3625 images. About 10 images from each category in the test set, for a total of 871 images. In this paper, 88 different types of seed images were collected, the largest species being legumes with 24 species, followed by gramineae with 22 species. The distribution of families and genera is shown in Fig. 6. As a seed dataset, this data set can be used not only for seed image classification in computer vision, but also as an important resource in seed image databases in agriculture. Figure 7 shows the display of seed images in the dataset.

## Technical Validation

In this section, we present an evaluation of advanced computer vision techniques as an initial benchmark for future work on LZUPSD^26^. For these fine-grained recognition related challenges, we employ several classical and state-of-the-art methods.

### Experimental setting

We divide each seed images into a training set and a testing set in a ratio of 80% and 20%. Then use the already divided training and testing sets in all experiments. We adopt PyTorch^27^ as our experiment framework based on a GPU platform with Nvidia RTX 4090. The size of the input image is 224 × 224, and the number of fully connected layers of the network is adjusted to 88, which matches the number of seed classes. Data enhancement is performed using random rotations and horizontal flips during the training phase. The optimizer uses Adam and has a learning rate of 0.0001. The batch size is set to 128, and each experiment is trained for 100 epochs. The loss function adopts cross entropy loss function.

### Evaluation index

The training set is used for training and the test set is used to evaluate model performance. In order to evaluate the recognition performance of each model, Accuracy, Precision and Recall are used as evaluation indexes in this experiment, and the formula for calculating the indexes is as follows:

1 $$Accuracy=\frac{TP+TN}{TP+TN+FP+FN}$$

2 $$Precision=\frac{TP}{TP+FP}$$

3 $$Recall=\frac{TP}{TP+FN}$$

### Analysis of network parameters

In the development process of deep learning, convolutional neural networks have shown excellent performance, and the Transformer model that has emerged in recent years has also shown amazing performance in fields such as image recognition. To verify that our dataset is suitable for training deep learning models, we conducted experiments using various mainstream classification models. Due to the significant impact of hyperparameter settings on image classification results, referring to previous researchers’ setting of model hyperparameters, we further conducted experiments on the selection of hyperparameters for the dataset LZUPSD. Specifically, the number of epoch is set to 100 because it can make the model converge. The training batch size is set to 128. Generally speaking, when supported by hardware devices, the larger the parameter, the better. The larger the training batch size, the more data the model can process at once. Adjusting the learning rate of the training optimizer based on the training batch size will achieve better results. To ensure fairness in all experiments, we directly use Adam as the optimizer. All experiments use cross entropy loss as the loss function. Regarding the selection of learning rate, we referred to the parameter settings of some classification models and conducted ablation experiments on the dataset LZUPSD. As shown in Table 2, two convolutional neural networks ResNet18^28^, DenseNet121^29^, and a Transformer based model ViT_B_16^30^ are selected for the experiments. The learning rate is set to 0.0001, which is slightly higher than 0.001. The reason why the experiment did not continue to compare and analyze the situation with lower learning rates is that when the learning rate is lower, the performance does not improve because the learning rate is too low and the model parameter optimization is too slow, resulting in the model being unable to converge within 100 epochs. The experiment found that pre training models on public large-scale datasets and then using the trained models as pre training model parameters can enhance the performance of the models. As shown in Table 3, we conducted ablation analysis on the impact of adding pre trained models on model performance. The dataset used for training the pre trained model is ImageNet^1^. Among them, Y represents loading the pre trained model, N represents not loading the pre trained model, and the initialization parameters are random. From Table 3, it can be seen that adding pre trained models greatly enhances model performance, especially for ViT models^30^. This is because compared to convolutional neural networks, Transformer require more datasets to achieve good performance.

**Table 2:** Ablation experiment on learning rate selection.

Model	Epoch	Batch Size	Pre Training	Optimizer	Learning Rate		Accuracy
					0.001	0.0001	
ResNet18^28^	100	128	✓	Adam		✓	92.8%
	100	128	✓	Adam	✓		91.4%
DenseNet121^29^	100	128	✓	Adam		✓	93.6%
	100	128	✓	Adam	✓		92.3%
ViT_B_16^30^	100	128	✓	Adam		✓	96.5%
	100	128	✓	Adam	✓		96.1%

**Table 3:** The impact of loading pre trained models on performance.

Model	Epoch	Batch Size	Learning Rate	Optimizer	Pre Training		Accuracy
					Y	N	
ResNet18^28^	100	128	0.0001	Adam		✓	86.8%
	100	128	0.0001	Adam	✓		92.8%
DenseNet121^29^	100	128	0.0001	Adam		✓	88.3%
	100	128	0.0001	Adam	✓		93.6%
ViT_B_16^30^	100	128	0.0001	Adam		✓	81.4%
	100	128	0.0001	Adam	✓		96.5%

We conducted a summary analysis of the results of some models tested on the dataset LZUPSD. As shown in Table 4, the training parameter settings and accuracy of ResNet series^28^, DenseNet series^29^, ViT series^30^, and TransFG^31^ models are listed in the table. Params is the parameter quantity of the model, Flops is the computational quantity of the model, where the computational quantity of the model is calculated when the input image size is 3 × 224 × 224.

**Table 4:** Comprehensive analysis of test results from multiple models.

Model	Epoch	Batch Size	Learning Rate	Optimizer	Pre Training	Accuracy	Params	Flops
ResNet18^28^	100	128	0.0001	Adam	✓	92.8%	11.22 M	1823.57 M
ResNet34^28^	100	128	0.0001	Adam	✓	93.1%	21.33 M	3678.27 M
ResNet50^28^	100	128	0.0001	Adam	✓	93.2%	23.69 M	4131.87 M
ResNet101^28^	100	128	0.0001	Adam	✓	94.1%	42.68 M	7864.57 M
DenseNet121^29^	100	128	0.0001	Adam	✓	93.6%	7.04 M	2896.07 M
DenseNet161^29^	100	128	0.0001	Adam	✓	94.7%	26.67 M	7843.83 M
DenseNet201^29^	100	128	0.0001	Adam	✓	94.5%	18.26 M	4388.63 M
ViT_B_16^30^	100	128	0.0001	Adam	✓	96.5%	57.37 M	11285.55 M
ViT_B_32^30^	100	128	0.0001	Adam	✓	95.1%	59.14 M	2950.67 M
ViT_L_16^30^	100	128	0.0001	Adam	✓	96.7%	202.43 M	39855.11 M
ViT_L_32^30^	100	128	0.0001	Adam	✓	95.8%	204.79 M	10230.60 M
TransFG^31^	100	128	0.0001	Adam	✓	96.2%	85.71 M	15574.25 M

### Fine-grained recognition

To verify the availability of data, several common neural networks are used for validation and to verify the availability of data and explore its effect on the task of seed classification. Compared with machine learning, convolutional neural network does not need to manually extract features, and has a strong learning ability, which can better realize the classification of seed images. The experimental methods and results are shown in Table 5. Among these methods, Resnet50^28^ is one of the most classical models, EfficientNet-b4^32^ is an advanced fine-grained recognition method, and TransFG^31^ is based on the popular transformer technique.

**Table 5:** Quantitative comparison of different methods on the test set.

Methods	Accuracy	Precision	Recall
Resnet50^28^	93.2%	93.8%	93.3%
DenseNet201^29^	94.5%	95.0%	94.7%
MobileNet_V2^35^	95.5%	96.0%	95.4%
SENet^33^	95.1%	95.7%	95.0%
BCNN^34^	93.0%	93.6%	93.0%
EfficientNet-b4^32^	95.6%	96.1%	95.5%
ViT_B_16^30^	96.5%	96.5%	96.6%
TransFG^31^	96.2%	96.6%	96.3%

The experimental results are shown in Table 5. The results show that the neural network can accurately identify the seed image. As the most commonly used network, ResNet^28^ is often used as the backbone network or for data set testing and other work. ResNet50 achieved 93.2% accuracy in the seed classification dataset. DenseNet201^29^ achieved 1.3% higher results than the ResNet50 network, and also had good accuracy and recall rates of 95.0% and 94.7%. The bilingual model and attention model commonly used in fine-grained recognition have remarkable effects. The attention network SENet^33^ and the bilingual model BCNN^34^ obtain better results than the general network. We used ResNet50 and SE modules to build the model with significantly higher accuracy than using resnet50 alone. SENet’s accuracy on the seed dataset reached 95.1%, SENet’s accuracy was 1.9% higher than ResNet50, and the accuracy and recall rate also improved by 1.9% and 1.7%. Experimental results show that SENet can focus the model’s attention on the seed part of the seed image through the SE module, which can significantly improve accuracy. The results show that the accuracy of seed classification can be effectively improved by the attention model. BCNN, which uses ResNet18 as its backbone network, achieves accuracy over ResNet50 through bilinear pooling. BCNN enriches the combination of features through bilinear pool, which enriches features and improves accuracy. The lightweight networks showed remarkable results, with the accuracy of 95.6% EfficientNet^32^ on the seed dataset. As a lightweight network, MobileNet_v2^35^ requires a relatively small amount of computation, but the accuracy rate is 95.5% and the recall rate is 96.0%. EfficientNet acts as a lightweight network, but achieves good results thanks to the balanced resolution of the images thanks to the depth and width of the models. Both lightweight models perform well on the seed classification dataset, particularly EfficientNet, achieving the highest accuracy. Lightweight network computing quantity is small, fast, can be made to go out to investigate in agriculture, hardware conditions are limited, and can still maintain a certain accuracy. In contrast to convolutional networks, Transformer requires a lot of data to be effective, and TransFG^31^ achieves a high accuracy of 96.2% by embedding pre-trained models.

### Visualization results and analysis

**Fig. 8:**
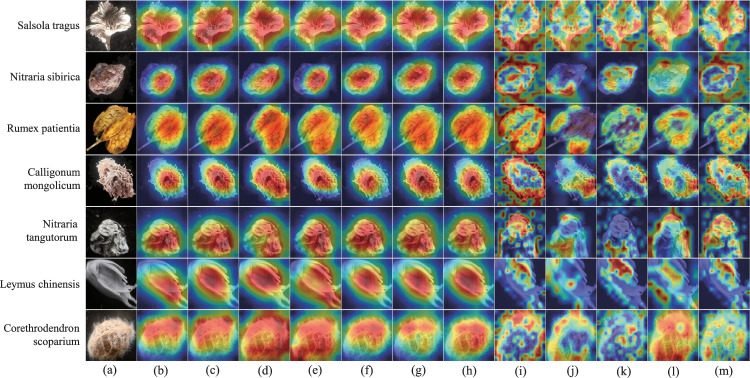
Model visualization results. (**a**) Original seed image, (**b**) Heat maps of ResNet18^28^, (**c**) Heat maps of ResNet34^28^, (**d**) Heat maps of ResNet50^28^, (**e**) Heat maps of ResNet101^28^, (**f**) Heat maps of DenseNet121^29^, (**g**) Heat maps of DenseNet161^29^, (**h**) Heat maps of DenseNet201^29^, (**i**) Heat maps of ViT_B_16^30^, (**j**) Heat maps of ViT_B_32^30^,(**k**) Heat maps of ViT_L_16^30^, (**l**) Heat maps of ViT_L_32^30^, (**m**) Heat maps of TransFG^31^.

Grad CAM (Gradient weighted Class Activation Mapping)^36^ is a technique that visualizes which parts of a deep neural network contribute the most to the prediction results. It can locate specific image regions, making the decision-making process of neural networks more interpretable and visual. In neural networks, the output feature map of the last convolutional layer has the greatest impact on the classification results. Therefore, we can calculate the weight of each channel by globally averaging pooling the gradient of the last convolutional layer. These weights can be used to weight the feature map and generate a Class Activation Map (CAM), where each pixel represents the importance of that pixel region to the classification results. This article uses Grad CAM to visualize various models to demonstrate their rationality. As shown in Fig. 8, each row represents an original seed image and visualized results of multiple models, while each column represents visualized results of different seeds under the same model. The models used in (b), (c), (d), (e), (f), (g), and (h) are convolutional neural networks, while (i), (j), (k), (l), and (m) are Transformers. It can be seen that there is a significant difference in the visual effect between the two. The former pays more attention to concentrated areas, while the latter pays more attention to scattered areas.

**Fig. 9:**
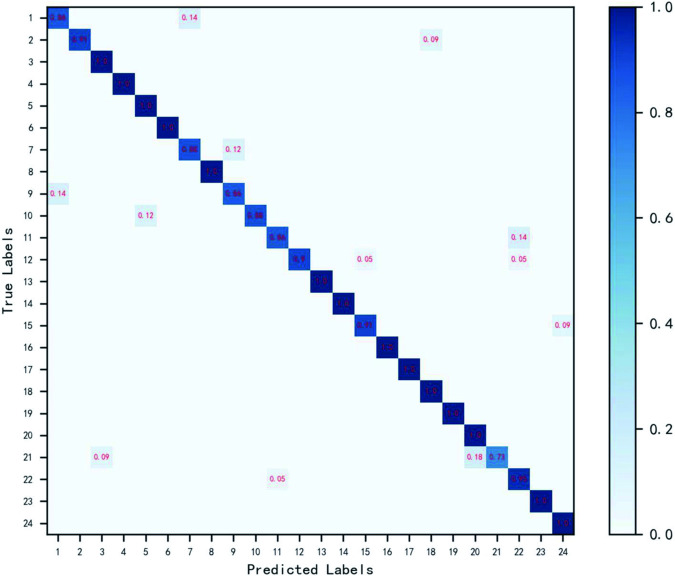
Confusion matrix of legume seeds. The horizontal axis is the predicted label of the seed, and the vertical axis is the true label of the seed. The data on the diagonal in the figure is correctly classified. The darker the color, the larger the value. The closer the value is to 1.0, the more accurate the classification is.

**Fig. 10:**
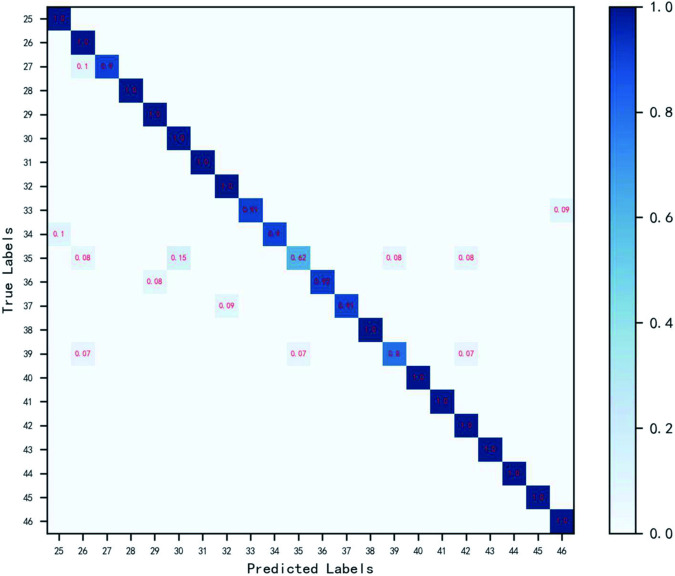
Confusion matrix of gramineous seeds. The horizontal axis is the predicted label of the seed, and the vertical axis is the true label of the seed. The data on the diagonal in the figure is correctly classified. The darker the color, the larger the value. The closer the value is to 1.0, the more accurate the classification is.

In order to study the classification effect of the seeds of the same family, separate experiments were carried out on the legume and gramineae, which have more species, in order to discuss the classification results of the seeds of the same family. The experiment uses EfficientNet^32^ as the classification model to classify them. Confusion matrices were drawn for grammar and grammar respectively to display the recognition results of grammar and grammar more intuitively. Figures 9, 10 show the confusion matrices of the two kinds of seeds. Table 1 lists the corresponding names. The diagonals in the figure are correctly classified. The darker the color, the greater the value. It can be seen from the figure that the data is centrally distributed on the diagonal, which proves that the model can well identify the seeds of the two families.

Among legumes, the seeds of Medicago sativa L and Melilotus officinalis Desr are similar in shape, color, texture, and are difficult to distinguish. However, the seeds of both species can be effectively distinguished using convolutional neural networks. Based on the observation of the confusion matrix in Figs. 9, 10, the accuracy of gramineous reed grass was comparatively low. Looking at the image, you can see that the seed shape is slender. Due to the growth period, shooting angle, preprocessing and other reasons, the shape of the seeds changes relatively, and the images of some seeds become relatively short and thicker, which makes the accuracy of the seeds low. In legumes, the accuracy of mongoliaceae is low. The seed shape may be slightly different in the image, but this is not the main reason for its low accuracy. Through observation, it was found that the color of the seeds produced a great difference. Due to the smooth surface of the seeds, the color of the seeds in the image may be changed due to the obvious change in the light angle during the growth period and shooting, which may be the main reason for the low accuracy. At the same time, during the experiment, it was found that the texture of some seeds would be damaged in the production and storage process. For example, some seeds have small fluff on the surface, which would inevitably wear out in the case of transportation, storage and filming, etc., which would also have a certain impact on the classification accuracy of seeds. Through the analysis of the identification results of the above seeds, it was found that the shape, color and texture of the seeds had a great influence on the results of seed classification. Therefore, in the process of storage, shooting and pretreatment, it is necessary to preserve these three features to the maximum extent to ensure accurate identification of seeds.

## Usage Notes

One of the great benefits of the LZUPSD database is that it is intended to help seed technologists and educators with the identification of different plant seed types by photos of the seeds. The new seed database created by us will be a critical tool to aid their efforts, helping inspectors make tough and tricky seed identifications and try to stop the entry and spread of noxious weeds into the country. It is also created for seed identification that can be used to train various models to extract morphological characteristics of seeds. Using a convolutional neural network model, seed image identification can be achieved, and the application of computer vision technology can be expanded in the grassland industry. This development can greatly benefit the grassland industry, leading to savings in both human and material resources, while promoting intelligent and modern agricultural practices.

## Data Availability

No custom code has been used.

## References

[1] Deng, J. *et al*. Imagenet: A large-scale hierarchical image database. In *2009 IEEE conference on computer vision and pattern recognition*, 248–255 (Ieee, 2009).

[2] Krause, J., Stark, M., Deng, J. & Fei-Fei, L. 3d object representations for fine-grained categorization. In *Proceedings of the IEEE international conference on computer vision workshops*, 554–561 (2013).

[3] Maji, S., Rahtu, E., Kannala, J., Blaschko, M. & Vedaldi, A. *Fine-grained visual classification of aircraft*. *arXiv preprint arXiv:1306.5151* (2013).

[4] Wah, C., Branson, S., Welinder, P., Perona, P. & Belongie, S. The caltech-ucsd birds-200-2011 dataset. (2011).

[5] Goëau, H., Bonnet, P. & Joly, A. Overview of lifeclef plant identification task 2020. In *CLEF 2020-Conference and labs of the Evaluation Forum*, vol. 2696 (2020).

[6] Quoc, T. N. & Hoang, V. T. Vnplant-200–a public and large-scale of vietnamese medicinal plant images dataset. In *Integrated Science in Digital Age 2020*, 406–411 (Springer, 2021).

[7] Sun, Y., Liu, Y., Wang, G. & Zhang, H. Deep learning for plant identification in natural environment. *Computational intelligence and neuroscience* **2017** (2017). 10.1155/2017/7361042 PMC5458433 28611840

[8] Tajbakhsh N (2020). Embracing imperfect datasets: A review of deep learning solutions for medical image segmentation. Medical Image Analysis.

[9] Sun, P. *et al*. Scalability in perception for autonomous driving: Waymo open dataset. In *Proceedings of the IEEE/CVF conference on computer vision and pattern recognition*, 2446–2454 (2020).

[10] Kamilaris A, Prenafeta-Boldú FX (2018). Deep learning in agriculture: A survey. Computers and electronics in agriculture.

[11] Zhou Q (2021). Geographical distribution of the dispersal ability of alien plant species in china and its socio-climatic control factors. Scientific Reports.

[12] Gao R, Li Q, Wu H, Lu F (2022). Salient regions and hierarchical indexing for crop disease images. International Journal of Pattern Recognition and Artificial Intelligence.

[13] Granitto PM, Navone HD, Verdes PF, Ceccatto H (2002). Weed seeds identification by machine vision. Computers and Electronics in agriculture.

[14] Granitto PM, Verdes PF, Ceccatto HA (2005). Large-scale investigation of weed seed identification by machine vision. Computers and Electronics in Agriculture.

[15] Luo, T. *et al*. Classification of weed seeds based on visual images and deep learning. *Information Processing in Agriculture* (2021).

[16] Giselsson, T. M., Jørgensen, R. N., Jensen, P. K., Dyrmann, M. & Midtiby, H. S. A public image database for benchmark of plant seedling classification algorithms. *arXiv preprint arXiv:1711.05458* (2017).

[17] Yang L (2022). Real-time classification of invasive plant seeds based on improved yolov5 with attention mechanism. Diversity.

[18] China Plant BOL Group (2011). Comparative analysis of a large dataset indicates that internal transcribed spacer (its) should be incorporated into the core barcode for seed plants. Proceedings of the National Academy of Sciences.

[19] You, Y. F. *et al*. Hazard status and control countermeasures of poisonous weeds in natural grasslands of inner mongolia. *Progress in Veterinary Medicine* (2018).

[20] Costanzo A, Bàrberi P (2014). Functional agrobiodiversity and agroecosystem services in sustainable wheat production. a review. Agronomy for Sustainable Development.

[21] Long, T. Present situation,problems and countermeasures of yingkou city vegetable seed industry development. *Horticulture & Seed* (2016).

[22] Turkoglu M, Aslan M, Ar A, Alin ZM, Hanbay D (2021). A multi-division convolutional neural network-based plant identification system. PeerJ Computer Science.

[23] Li, P. *et al*. Plant identification based on multi-branch convolutional neural network with attention. In *Chinese Conference on Image and Graphics Technologies*, 472–481 (Springer, 2019).

[24] Golpour I (2014). Identification and classification of bulk paddy, brown, and white rice cultivars with colour features extraction using image analysis and neural network. Czech Journal of Food Sciences.

[25] Guzman, J. D. *et al*. Classification of philippine rice grains using machine vision and artificial neural networks. In *World conference on Agricultural information and IT*, vol. 6, 41–48 (2008).

[26] Yuan M (2023). figshare.

[27] Paszke, A. *et al*. Pytorch: An imperative style, high-performance deep learning library. *Advances in neural information processing systems* **32** (2019).

[28] He, K., Zhang, X., Ren, S. & Sun, J. Deep residual learning for image recognition. In *Proceedings of the IEEE conference on computer vision and pattern recognition*, 770–778 (2016).

[29] Huang, G., Liu, Z., Van Der Maaten, L. & Weinberger, K. Q. Densely connected convolutional networks. In *Proceedings of the IEEE conference on computer vision and pattern recognition*, 4700–4708 (2017).

[30] Dosovitskiy, A. *et al*. An image is worth 16 × 16 words: Transformers for image recognition at scale. *arXiv preprint arXiv:2010.11929* (2020).

[31] He J (2022). Transfg: A transformer architecture for fine-grained recognition. Proceedings of the AAAI Conference on Artificial Intelligence.

[32] Tan, M. & Le, Q. Efficientnet: Rethinking model scaling for convolutional neural networks. In *International conference on machine learning*, 6105–6114 (PMLR, 2019).

[33] Hu, J., Shen, L. & Sun, G. Squeeze-and-excitation networks. In *Proceedings of the IEEE conference on computer vision and pattern recognition*, 7132–7141 (2018).

[34] Lin, T.-Y., RoyChowdhury, A. & Maji, S. Bilinear cnn models for fine-grained visual recognition. In *Proceedings of the IEEE international conference on computer vision*, 1449–1457 (2015).

[35] Howard, A. G. *et al*. Mobilenets: Efficient convolutional neural networks for mobile vision applications. *arXiv preprint arXiv:1704.04861* (2017).

[36] Selvaraju, R. R. *et al*. Grad-cam: Why did you say that? *arXiv preprint arXiv:1611.07450*. 10.48550/arXiv.1611.07450 (2016).

